# Effect of Advanced Shoe Technology on the Evolution of Road Race Times in Male and Female Elite Runners

**DOI:** 10.3389/fspor.2021.653173

**Published:** 2021-04-22

**Authors:** Stéphane Bermon, Frédéric Garrandes, Andras Szabo, Imre Berkovics, Paolo Emilio Adami

**Affiliations:** ^1^Health and Science Department, World Athletics, Manoco City, Monaco; ^2^Laboratoire Motricité Humaine Expertise Sport Santé, Université Côte d'Azur, Nice, France; ^3^Elite Limited, Budapest, Hungary; ^4^Department of Movement, Human and Health Sciences, University of Rome “Foro Italico,” Rome, Italy

**Keywords:** athletics, footwear, gender, marathon, performance

## Abstract

The influence of advanced footwear technology (thickness of light midsole foam and rigid plate) on distance running performances was analyzed during an 8-year period. Analysis of variance was used to measure effects of time, gender, shoe technology, and East African origin on male and female top 20 or top 100 seasonal best times in 10-kilometer races, half-marathons, and marathons. In both genders and three distance-running events, seasonal best times significantly decreased from 2017, which coincided with the introduction of the advanced footwear technology in distance running. This performance improvement was of similar magnitude in both East African and non-East African elite runners. In female elite athletes, the magnitudes (from 1.7 to 2.3%) of the decrease in seasonal best times between 2016 and 2019 were significantly higher than in their male counterparts (from 0.6 to 1.5%). Analyses of variance confirmed that the adoption of the advanced footwear technology significantly improved the top 20 seasonal best times in female half marathons and marathons and male marathons, with the improvements being more pronounced in females and in long-distance running events. The adoption of this new shoe technology improved female marathon time by ~2 min and 10 s, which represents a significant increase in performance (1.7%).

## Introduction

In 2017, Nike officially presented its Nike® Vaporfly 4% shoes. The release was associated with a large and worldwide advertising campaign that received a lot of attention because the manufacturer claimed that this new model of distance running shoes represented a breakthrough in the distance running shoe technology that could improve running time by 4% (Hoogkamer et al., 2018). This advanced footwear technology (AFT) relies on the combination of a very thick and light midsole made of polyamide block elastomer and the embedding within the midsole layers of a long and rigid carbon plate. What was initially considered as a marketing maneuver by the manufacturer quickly turned to a possible game changer in the world of distance running performance. Although several theories (Nigg et al., 2020b; Cigoja et al., 2021; Muniz-Pardos et al., 2021) have been proposed to understand the mechanisms behind these performance benefits, no definitive explanation has been provided so far. A large-scale statistical analysis published in 2018 (Quealy and Katz, 2018), based on results from 280,000 marathons and 215,000 half-marathons run, revealed that this AFT was likely responsible for a 3–4% decrease in race times in these distances. Although these statistics are based on large numbers, the authors acknowledged some limitations in their study as it was based on athletes' self-declaration on an app dedicated to tracking running and cycling activities and did not specifically address elite distance runners. Moreover, the male and female half-marathon and marathon world records were all broken in 2018 and 2019 by Ethiopian and Kenyan athletes using AFT (Hoogkamer et al., 2017). Altogether, these facts fueled the controversy about the performance advantage (Muniz-Pardos et al., 2021), which some believe contradicts World Athletics' technical rule 143, paragraph 5.2, which states (World Athletics, 2020) that “athletes may compete barefoot or with footwear on one or both feet. The purpose of shoes for competition is to give protection and stability to the feet and a firm grip on the ground. They must not give athletes any unfair assistance or advantage. Any type of shoe must be reasonably available to all in the spirit of the universality of athletics.…” Therefore, the purpose of this exploratory study is to analyze elite male and female runners' official race results recorded between 2012 and 2019 in the 10-kilometer, half-marathon and marathon races. We tested the hypotheses that after 2017, elite runners will show relative decreases in race times such as those observed in sub-elite and club runners and that such decreases will be explained by the adoption of AFT by elite distance runners.

## Methods and Materials

The evolution of elite male and female distance runner race times between 2012 and 2019 (included) were assessed in three different road race events: the 10 kilometers (10 km), half-marathon, and marathon. For each of the 8 years studied, top 20 and top 100 individual seasonal (yearly) best performances for both genders were used for the purpose of statistical analysis. Competition results not validated by World Athletics or obtained from athletes disqualified because of anti-doping rules violations committed during the competition considered were excluded from the analysis. Results were obtained from World Athletics' official database. On the basis of declared citizenship, federation information on transfer of allegiance, and biographies, athletes from Djibouti, Ethiopia, Eritrea, Kenya, Rwanda, Uganda, Somalia, and Tanzania were grouped in an “East African” group, whereas the other athletes formed the “non-East African” group. For each gender and each event, the evolution of the top 20 and top 100 individual seasonal best times was explored with a one-way ANOVA (year) or two-way ANOVA (year^*^ethnic group or year^*^gender) and, when appropriate, Bonferroni-corrected for multiple-comparison *post-hoc* tests and Cohen's *d*. As the AFT was available early 2017, we searched when this technology was adopted by each of the top 20 male and female athletes in the three events between 2017 and 2019. As this search was performed from analysis of media content, photos, and footage of athletes in competition, it was impossible to perform it for the top 100 seasonal best times. We assumed that contracted athletes always competed with an unmodified model of shoes provided by his/her partner manufacturer. With this information, the effects of the adoption of AFT, gender, and running events on the top 20 seasonal best times were explored with one-, two-, or three-way ANOVA and, when appropriate, Bonferroni-corrected for multiple-comparison *post-hoc* tests. When the same athletes achieved performances between 2016 and 2019, with and without the AFT, their results were compared by using a paired *t* test after a normality check with the Shapiro-Wilk test. Descriptive statistics are presented with the mean and standard deviation. Statistical significance was considered to be indicated by a *p*-value < 0.05. Statistical analyses were performed using the JASP 0.13.1 free statistical software.

## Results

### Top 100 Seasonal Best Times

The evolution of the top 100 male and female seasonal best times in the three events we have discussed is presented in Table 1. In both genders—and for the three distance running events—a decrease of mean seasonal best times was observed from 2017, with the lowest race times being recorded in 2019. The effect of time was significantly different between males and females—*F*_(1,7)_ = 6.53, 10.69, 8.60, *p* < 0.001—in the 10-km, half-marathon, and marathon top 100 seasonal best times, respectively. When we pooled the three distance running events, non-East African athletes represented on average 13% (range: 5–32%) and 28% (range: 12–44%) of the studied male and female populations, respectively. In all distance running events, the evolution of the top 100 seasonal best times followed a similar pattern across the years among East African and non-East African male (*F* = 0.49, *p* = 0.94) and female (*F* = 0.58, *p* = 0.88) athletes. As shown in Table 1, female athletes demonstrated larger decreases in race times between 2016 and 2019 than their male counterparts.

**Table 1:** Evolution of top 100 male and female seasonal best times in the 10 km, half-marathon, and marathon races.

	**Year**								
	**2012**	**2013**	**2014**	**2015**	**2016**	**2017**	**2018**	**2019**	***F*_(7,792)_,** ***p*-values**
**Male events**									
10 km [min:s]	28:13.8 (00:14.7)	28:07.4 (00:14.7)	28:14.3 (00:16.9)	28:14.1 (00:16.4)	28:19.2 (00:17.8)	28:13.4 (00:19.2)	28:07.9 (00:23.6)^***^ †	27:59.8 (00:22.3)^***^ ‡	10.36, *p* < 0.001
Half Marathon [h:min:s]	01:00:33.8 (00:43.0)	01:00:38.7 (00:37.1)	01:00:21.7 (00:39.0)	01:00:14.9 (00:28.9)	01:00:31.9 (00:42.1)	01:00:18.5 (00:37.3)	01:00:07.0 (00:34.6)^***^ †	01:00:05.3 (00:37.5)^***^ †	10.85, *p* < 0.001
Marathon [h:min:s]	02:06:58.4 (01:12.4)	02:07:33.0 (01:22.5)	02:07:19.1 (01:18.4)	02:07:42.5 (01:08.0)	02:07:42.1 (01:34.9)	02:07:27.9 (01:22.4)	02:06:54.1 (01:28.5)^***^ †	02:06:07.2 (01:20.4)^***^ ‡	15.70, *p* < 0.001
**Female events**									
10 km [min:s]	32:15.1 (00:28.6)	32:17.5 (00:26.0)	32:09.4 (00:26.5)	32:12.2 (00:29.0)	32:15.5 (00:33.2)	32:00.0 (00:38.0)	31:57.3 (00:33.1)^***^ †	31:39.4 (00:36.2)^***^ ‡	16.75 *p* < 0.001
Half-Marathon [h:min:s]	01:09:40.2 (00:01:15.2)	01:09:25.8 (01:11.2)	01:09:26.9 (01:00.7)	01:09:31.7 (01:07.9)	01:09:17.6 (01:20.3)	01:08:42.9 (01:25.2)	01:08:19.3 (01:23.3)^***^ †	01:08:05.1 (01:16.3)^***^ ‡	23.05 *p* < 0.001
Marathon [h:min:s]	02:24:58.2 (02:31.8)	02:25:54.6 (02:14.6)	02:26:27.8 (02:24.9)	02:25:32.4 (02:14.2)	02:25:40.3 (02:08.9)	02:25:05.8 (02:32.7)	02:23:46.7 (02:23.1)^***^ ‡	02:22:45.4 (02:14.9)^***^ ‡	26.55 *p* < 0.001

*Results are presented as mean (standard deviation)*.

*** *Different than 2016 race time: p < 0.001*.

† *Cohen's d > 0.5 (medium effect size)*.

‡ *Cohen's d > 0.8 (large effect size)*.

### Top 20 Seasonal Best Times

The evolution of the top 20 male and female seasonal best times in the three events is presented in Table 2. Our analysis showed that the AFT was adopted by a limited number of runners in 2017 when this technology had only been released. However, in 2018 and especially in 2019, many seasonal best times (the top 20) were achieved by athletes running with the AFT. In 2019, 55–95% of elite male and 45–80% of female runners used advanced footwear technology in 10 km, half-marathon, and marathon races. During the period studied, when gender and events results were pooled, the adoption of the AFT showed a significant performance-enhancing effect (*F* = 120.3, *p* < 0.001). This improvement of seasonal best times was more important in female athletes (*F* = 17.9, *p* < 0.001) and in longer distance running events (*F* = 31.03, *p* < 0.001; see Table 3). In half-marathon and marathon races, we were able to identify small numbers of athletes who competed in the same event within the 2016–2019 period with and without the AFT. All athletes (except male half-marathon runners) significantly improved their seasonal best times when using the AFT (Table 4).

**Table 2:** Evolution of top 20 male and female seasonal best times in the 10 km, half-marathon, and marathon races.

	**Year**								
	**2012**	**2013**	**2014**	**2015**	**2016**	**2017**	**2018**	**2019**	***F*_(7,152)_, *p*-values**
**Male events**									
10 km [min:s]	27:50.6 (00:08.9)	27:43.1 (00:07.4)	27:45.9 (00:11.3)	27:48.1 (00:07.0)	27:49.9 (00:9.6)	27:44.8 (00:16.1)	27:30.7 (00:14.5)^***^ ^†^	27:24.3 (00:16.4)^***^ ^†^	12.76, *p* < 0.001
Half Marathon [h:min:s]	59:23.3 (00:18.7)	59:39.5 (00:27.0)	59:21.7 (00:15.1)	59:30.1 (00:10.6)	59:27.7 (00:15.3)	59:20.6 (00:17.6)	59:11.8 (00:18.2)	59:07.7 (00:20.2)^*^ ^†^	6.03, *p* < 0.001
Marathon [h:min:s]	02:05:02.1 (00:28.7)	02:05:16.3 (00:58.7)	02:05:14.3 (01:03.2)	02:05:56.0 (00:38.7)	02:05:08.0 (01:06.2)	02:05:20.9 (00:49.4)	02:04:33.0 (00:51.8)	02:03:59.5 (00:57.6)^**^ ^†^	8.48, *p* < 0.001
**Female events**									
10 km [min:s]	31:29.7 (00:21.4)	31:38.1 (00:28.5)	31:26.0 (00:19.9)	31:25.3 (00:25.9)	31:23.1 (00:23.9)	30:59.7 (00:28.9)	31:05.3 (00:21.8)	30:41.3 (00:24.4)^***^ ^†^	11.82, *p* < 0.001
Half-Marathon [h:min:s]	01:07:46.0 (00:33.0)	01:07:32.6 (00:42.1)	01:07:59.3 (00:53.9)	01:07:42.7 (01:00.0)	01:07:10.2 (00:45.8)	01:06:26.8 (00:51.2)	01:06:09.6 (00:43.7)^**^ ^†^	01:06:03.2 (00:23.4)^***^ ^†^	20.97, *p* < 0.001
Marathon [h:min:s]	02:20:49.0 (01:10.5)	02:22:31.1 (01:13.9)	02:22:30.9 (01:35.4)	02:22:15.4 (01:27.1)	02:22:30.9 (01:12.3)	02:20:57.4 (01:26.1)^*^ ^†^	02:20:00.3 (01:13.6)^***^ ^†^	02:19:18.2 (01:38.3)^***^ ^†^	17.02, *p* < 0.001

*Results are presented as mean (standard deviation)*.

* *Different than 2016 race time: p < 0.05*,

** *Different than 2016 race time: p < 0.01*.

*** *Different than 2016 race time: p < 0.001*.

† *Cohen's d > 0.8 (large effect size)*.

**Table 3:** Effects of the advanced footwear technology on top 20 male and female seasonal best times in 10 km, half marathon, and marathon.

	**No AFT**	**AFT**	***t*, *p*-values**	**Cohen's *d***
**Male events**				
10 km [min:s]	27:44.7 (00:13.6) *n* = 130	27:30.7 (00:14.8)*n* = 28	4.85, *p* < 0.001	1.01
Half-Marathon [h:min:s]	59:24.8 (00:19.5) *n* = 140	59:09.2 (00:21.2)*n* = 20	3.32, *p* < 0.01	0.79
Marathon [h:min:s]	02:05:18.3 (00:53.6) *n* = 123	02:04:15.4 (01:09.3)*n* = 37	6.06, *p* < 0.001	1.14
**Female events**				
10 km [min:s]	31:18.4 (00:30.1) *n* = 136	31:01.3 (00:23.8)*n* = 23	2.58, *p* < 0.05	0.58
Half Marathon [h:min:s]	01:07:15.9 (01:02.2) *n* = 135	01:06:14.5 (00:29.4)*n* = 25	4.83, *p* < 0.001	1.05
Marathon [h:min:s]	02:21:49.9 (01:34.9) *n* = 124	02:19:39.4 (01:30.6)*n* = 35	7.25, *p* < 0.001	1.39

*Results are presented as mean (standard deviation)*.

*AFT, advanced footwear technology*.

**Table 4:** Comparison of male and female seasonal best times in half-marathon and marathon races in a subgroup of top 20 athletes who competed without and with the AFT between 2016 and 2019.

	**Without AFT**	**With AFT**	***t*, *p*-values**	**Cohen's *d***
**Male events**				
Half-Marathon [h:min:s]*n* = 7	59:08.4 (00:23.4)	59:00.3 (00:26.6)	0.61, NS	/
Marathon [h:min:s]*n* = 8	02:05:07.4 (01:20.6)	02:03:25.6 (01:09.9)	2.70, *p* < 0.05	1.35
**Female events**				
Half Marathon [h:min:s]*n* = 6	01:07:04.2 (00:41.4)	01:05:55.4 (00:30.0)	3.29, *p* < 0.01	1.90
Marathon [h:min:s]*n* = 6	02:21:56.5 (01:06.7)	02:18:55.9 (02:55.9)	2.35, *p* < 0.05	1.36

*Results are presented as mean (standard deviation)*.

*AFT, advanced footwear technology*.

## Discussion

The main result from this retrospective study is a significant decrease in elite athletes' seasonal bests times in 10 km, half marathon and marathon races for both genders from 2017. This change coincides with the release of the AFT and its adoption by elite athletes, and it has itself been identified as a factor improving seasonal best times. A decrease of seasonal bests has been found at the highest elite (top 20) and elite (top 100) levels. The reported decreases in race times observed from 2016 (female marathon) and 2017 (all others) is unlikely to be explained by a decennial trend since performances were rather steady between 2012 and 2016 in both genders and in the three distances. The fact that the top 20 seasonal best times do not always reflect performances of the same people from year to year is a limitation of the present study. Indeed, in view of the duration of the period studied and the high “turn-over rate” of East African elite-level athletes in road running events, it was almost impossible to set up a large enough dataset on which a robust repeated-measures analysis of variance could be conducted. However, our complementary analysis conducted on limited numbers of half-marathon and marathon runners for whom we had seasonal best times achieved with and without the AFT tend to confirm the results reported for top 20 and top 100 groups as well as the performance-enhancing effect of the AFT.

Low marathon race times observed in male and female runners in 2012 are an unexpected result. This could be explained by a larger number of competitors abusing performance enhancing drugs in that year. In 2012, the International Association of Athletics Federations (IAAF now World Athletics) was indeed already running a solid in-competition testing program complemented by an athlete biological passport program (Saugy et al., 2014), but this new generation of anti-doping programs was in its early phases and improved over time. Interestingly, the top 1,000 seasonal best times in male and female marathon runners show (results not presented) that even sub-elite or high-level club runners achieved good results in 2012, making the doping hypothesis less likely. An alternative explanation may be related to environmental race conditions. NASA's Goddard Institute for Space Studies (NASA, 2020) reported that, on a global level, 2012 was cooler than the seven following years. As high air temperature and relative humidity are known to limit endurance performance (Maughan, 2010), slightly cooler conditions encountered in 2012, could have facilitated achievement of better results in marathon competitions. Missing or unavailable data on weather for most races considered is a limiting factor in our study. However, the fact that seasonal best times recorded during the 8-year period were obtained from more than 200 different races organized in the spring and fall of both hemispheres makes it very unlikely that thermal stress accounts for the observed improvement in road race performances.

While looking for evidence of AFT adoption in the top 20 runners, we noted that some of them later committed anti-doping rules violations and subsequently served a period of ineligibility. Although such cheating behavior could represent a bias, we believe that it is unlikely to explain the observed performance improvements after 2017. Indeed, information obtained from the Athletics Integrity Unit website (Athletics Integrity Unit, 2020), shows that adverse passport findings and blood doping cases have not been reported to be more frequent in the period 2017–2019, compared with 2012–2016.

As shown in Tables 1, 2, the year 2017 was a turning point in the road running industry with the first release by a shoe manufacturer of a model of distance running shoe that benefited from an AFT incorporating both an increased thickness of a new midsole light foam and a rigid plate along the shoe (Nigg et al., 2020a). The present study is a retrospective observational study from which a possible causality link between the availability of this AFT and distance running performances in top 20 seasonal best times can be derived. Moreover, the market release of AFT and its progressive adoption by elite distance runners coincided with the observed significant trend in improved performances. An alternative explanation to our main finding would be a possible overrepresentation of East African runners at the highest level of endurance competition. Indeed, East African runners have dominated distance running in athletics for almost two decades (Tucker et al., 2013). Although our demographic data suggest such a trend in female athletes, this hypothesis can be discarded since non-East African elite male and female distance runners, while less numerous than East African runners, also significantly improved their performances from 2017.

The magnitude of seasonal best-time decreases was larger in elite females when compared with elite male runners. This phenomenon is also observed in the top 100 statistical analyses. Indeed, between 2016 and 2019, female race times decreased by 1.9, 1.7, and 2.0% in the 10 km, half-marathon, and marathon respectively, whereas these decreases were calculated at 1.1, 0.7, and 1.2% in male runners. In the consideration of top 20 seasonal bests, where the effect of AFT could be tested and quantified, the adoption of this new shoe technology improved female marathon time by ~2 min and 10 s. Such a 1.7% increase in performance is remarkable at the elite level. For purposes of comparison only, Malm et al. (2016) reported an average 3% performance increase after blood doping. The top 20 male and top 20 female runners adopted the AFT to a similar extent between 2017 and 2019. However, the race time decrease observed between 2016 and 2019 always appeared to be larger for females than for males. This would suggest that this technology benefits female athletes more than males. As women, when compared with men, show greater fatigue resistance, greater substrate efficiency, and lower energetic demands during endurance events (Hunter, 2016; Tiller et al., 2021), the female lower body mass and/or a smaller shoes sizes could represent a possible explanation for this gender difference. It could be hypothesized that smaller shoe size is associated with a shorter but stiffer rigid plate in the AFT (Hoogkamer et al., 2017), and/or a higher midsole thickness/body mass ratio, facilitating a higher percentage of energy return in female runners (Hoogkamer et al., 2018).

Although highly significant at this competition level, the magnitude of elite runners' race time decrease reported in this study is lower than the average 3.4% change of running velocity calculated by Hoogkamer et al. (2018) in male sub-elite runners in their comparison of AFT with classical footwear. In their study, the authors measured energy costs of running 5 min at 14, 16, and 18 km/h, and concluded that the percentage of savings was similar at the three velocities. These experimental conditions (only applied to male subjects) are somewhat different from real race conditions, where higher velocities are maintained from approximately 28 min (10 km) to 145 min (marathon). During this extended time, the energy cost of running may progressively increase, due to slow component increases in oxygen uptake kinetics (Jones et al., 2011) and muscle damage (Assumpcao Cde et al., 2013). The magnitude of elite runners' race time decreases reported in this study is also lower than the ~4% reported in club and sub-elite runners (Quealy and Katz, 2018).

## Conclusions

The present study showed that top 20 and top 100 seasonal best times in 10 km, half-marathons, and marathons significantly decreased from 2017. Adoption of the advanced footwear technology has been identified as a factor contributing to these observed changes. The magnitude of this relative change was higher in female than in male elite athletes and was more pronounced in marathons than in 10 km road races. Although very relevant at an elite level, it appears that the magnitude of elite runners' race time changes observed between 2016 and 2019 is lower than race time changes reported in club or sub-elite distance runners.

## Data Availability Statement

The race results raw data supporting the conclusions of this article will be made available by the authors, without undue reservation.

## Author Contributions

SB, FG, AS, IB, and PA contributed to the conception or design of the work, drafted the manuscript, and critically revised the manuscript. All gave final approval and agreed to be accountable for all aspects of work, ensuring integrity and accuracy.

## Conflict of Interest

AS and IB were employed by the company Elite Ltd, Budapest. The remaining authors declare that the research was conducted in the absence of any commercial or financial relationships that could be construed as a potential conflict of interest.
